# Supplementary material for: Multistate animal-contact-related nontyphoidal Salmonella enterica outbreaks in the United States, 2009–2022: Network and machine learning analyses of exposure sources, settings, and serovars
Source: PLoS One. 2026 Jun 5;21(6):e0344889. doi: 10.1371/journal.pone.0344889 (PMC13240899; doi:10.1371/journal.pone.0344889)
Supplement: S1 Table — The S1 Table represents the national NTS multistate outbreak incidence rates (Per 10 Million Population-Years). (DOCX) [file pone.0344889.s001.docx]

**S1 Table. National-level nontyphoidal *Salmonella enterica* animal contact-related multistate outbreak and illness incidence rates across the U.S. 2009 – 2022**

| **year** | **Outbreak Number** | **States mentioned** | **States involved** | **State Names** | **Outbreak IRs (Per 10MPY)** |
| --- | --- | --- | --- | --- | --- |
| 2009 | 7 | 87 | 46 | Alabama; Alaska; Arizona; California; Colorado; Connecticut; Florida; Georgia; Idaho; Illinois; Indiana; Iowa; Kansas; Kentucky; Louisiana; Maine; Maryland; Massachusetts; Michigan; Minnesota; Mississippi; Missouri; Montana; Nebraska; Nevada; New Hampshire; New Jersey; New Mexico; New York; North Carolina; Ohio; Oklahoma; Oregon; Pennsylvania; Rhode Island; South Carolina; South Dakota; Tennessee; Texas; Utah; Vermont; Virginia; Washington; West Virginia; Wisconsin; Wyoming | 0.10 |
| 2010 | 6 | 95 | 44 | Alabama; Alaska; Arizona; Arkansas; California; Colorado; Connecticut; Delaware; Florida; Georgia; Hawaii; Idaho; Illinois; Indiana; Iowa; Kansas; Kentucky; Maine; Maryland; Massachusetts; Michigan; Minnesota; Missouri; Nebraska; New Hampshire; New Jersey; New Mexico; New York; North Carolina; Ohio; Oklahoma; Oregon; Pennsylvania; Rhode Island; South Carolina; South Dakota; Tennessee; Texas; Utah; Vermont; Virginia; Washington; West Virginia; Wisconsin | 0.08 |
| 2011 | 12 | 186 | 48 | Alabama; Alaska; Arizona; Arkansas; California; Colorado; Connecticut; Delaware; District of Columbia; Florida; Georgia; Idaho; Illinois; Indiana; Iowa; Kansas; Kentucky; Louisiana; Maine; Maryland; Massachusetts; Michigan; Minnesota; Mississippi; Missouri; Montana; Nebraska; Nevada; New Hampshire; New Jersey; New Mexico; New York; North Carolina; Ohio; Oregon; Pennsylvania; Puerto Rico; Rhode Island; South Carolina; South Dakota; Tennessee; Texas; Utah; Vermont; Virginia; Washington; West Virginia; Wisconsin | 0.08 |
| 2012 | 15 | 237 | 51 | Alabama; Alaska; Arizona; Arkansas; California; Colorado; Connecticut; Delaware; Florida; Georgia; Hawaii; Idaho; Illinois; Indiana; Iowa; Kansas; Kentucky; Louisiana; Maine; Maryland; Massachusetts; Michigan; Minnesota; Mississippi; Missouri; Montana; Nebraska; Nevada; New Hampshire; New Jersey; New Mexico; New York; North Carolina; North Dakota; Ohio; Oklahoma; Oregon; Pennsylvania; Puerto Rico; Rhode Island; South Carolina; South Dakota; Tennessee; Texas; Utah; Vermont; Virginia; Washington; West Virginia; Wisconsin; Wyoming | 0.07 |
| 2013 | 4 | 100 | 47 | Alabama; Arizona; Arkansas; California; Colorado; Connecticut; Delaware; Florida; Georgia; Idaho; Illinois; Indiana; Iowa; Kansas; Kentucky; Louisiana; Maine; Maryland; Massachusetts; Michigan; Minnesota; Mississippi; Missouri; Montana; Nebraska; Nevada; New Hampshire; New Jersey; New Mexico; New York; North Carolina; North Dakota; Ohio; Oklahoma; Oregon; Pennsylvania; South Carolina; South Dakota; Tennessee; Texas; Utah; Vermont; Virginia; Washington; West Virginia; Wisconsin; Wyoming | 0.05 |
| 2014 | 6 | 105 | 46 | Alabama; Arizona; Arkansas; California; Colorado; Connecticut; Florida; Georgia; Idaho; Illinois; Indiana; Iowa; Kansas; Kentucky; Louisiana; Maine; Maryland; Massachusetts; Michigan; Minnesota; Mississippi; Missouri; Montana; Nebraska; Nevada; New Hampshire; New Jersey; New Mexico; New York; North Carolina; Ohio; Oklahoma; Oregon; Pennsylvania; Puerto Rico; South Carolina; South Dakota; Tennessee; Texas; Utah; Vermont; Virginia; Washington; West Virginia; Wisconsin; Wyoming | 0.07 |
| 2015 | 13 | 225 | 47 | Alabama; Arizona; Arkansas; California; Colorado; Connecticut; Delaware; Florida; Georgia; Hawaii; Idaho; Illinois; Indiana; Iowa; Kansas; Kentucky; Louisiana; Maine; Maryland; Massachusetts; Michigan; Minnesota; Mississippi; Missouri; Montana; Nevada; New Hampshire; New Jersey; New Mexico; New York; North Carolina; North Dakota; Ohio; Oklahoma; Oregon; Pennsylvania; South Carolina; South Dakota; Tennessee; Texas; Utah; Vermont; Virginia; Washington; West Virginia; Wisconsin; Wyoming | 0.06 |
| 2016 | 9 | 193 | 48 | Alabama; Alaska; Arizona; Arkansas; California; Colorado; Connecticut; Delaware; Florida; Georgia; Idaho; Illinois; Indiana; Iowa; Kansas; Kentucky; Louisiana; Maine; Maryland; Massachusetts; Michigan; Minnesota; Mississippi; Missouri; Montana; Nebraska; Nevada; New Hampshire; New Jersey; New York; North Carolina; North Dakota; Ohio; Oklahoma; Oregon; Pennsylvania; Rhode Island; South Carolina; South Dakota; Tennessee; Texas; Utah; Vermont; Virginia; Washington; West Virginia; Wisconsin; Wyoming | 0.06 |
| 2017 | 13 | 251 | 50 | Alabama; Arizona; Arkansas; California; Colorado; Connecticut; Delaware; District of Columbia; Florida; Georgia; Hawaii; Idaho; Illinois; Indiana; Iowa; Kansas; Kentucky; Louisiana; Maine; Maryland; Massachusetts; Michigan; Minnesota; Mississippi; Missouri; Montana; Nebraska; Nevada; New Hampshire; New Jersey; New Mexico; New York; North Carolina; North Dakota; Ohio; Oklahoma; Oregon; Pennsylvania; Rhode Island; South Carolina; South Dakota; Tennessee; Texas; Utah; Vermont; Virginia; Washington; West Virginia; Wisconsin; Wyoming | 0.06 |
| 2018 | 12 | 202 | 49 | Alabama; Alaska; Arizona; Arkansas; California; Colorado; Connecticut; Florida; Georgia; Hawaii; Idaho; Illinois; Indiana; Iowa; Kansas; Kentucky; Louisiana; Maine; Maryland; Massachusetts; Michigan; Minnesota; Mississippi; Missouri; Montana; Nebraska; Nevada; New Hampshire; New Jersey; New Mexico; New York; North Carolina; North Dakota; Ohio; Oklahoma; Oregon; Pennsylvania; Rhode Island; South Carolina; South Dakota; Tennessee; Texas; Utah; Vermont; Virginia; Washington; West Virginia; Wisconsin; Wyoming | 0.08 |
| 2019 | 17 | 349 | 50 | Alabama; Arizona; Arkansas; California; Colorado; Connecticut; Delaware; District of Columbia; Florida; Georgia; Hawaii; Idaho; Illinois; Indiana; Iowa; Kansas; Kentucky; Louisiana; Maine; Maryland; Massachusetts; Michigan; Minnesota; Mississippi; Missouri; Montana; Nebraska; Nevada; New Hampshire; New Jersey; New Mexico; New York; North Carolina; North Dakota; Ohio; Oklahoma; Oregon; Pennsylvania; Rhode Island; South Carolina; South Dakota; Tennessee; Texas; Utah; Vermont; Virginia; Washington; West Virginia; Wisconsin; Wyoming | 0.06 |
| 2020 | 27 | 585 | 51 | Alabama; Alaska; Arizona; Arkansas; California; Colorado; Connecticut; Delaware; District of Columbia; Florida; Georgia; Hawaii; Idaho; Illinois; Indiana; Iowa; Kansas; Kentucky; Louisiana; Maine; Maryland; Massachusetts; Michigan; Minnesota; Mississippi; Missouri; Montana; Nebraska; Nevada; New Hampshire; New Jersey; New Mexico; New York; North Carolina; North Dakota; Ohio; Oklahoma; Oregon; Pennsylvania; Rhode Island; South Carolina; South Dakota; Tennessee; Texas; Utah; Vermont; Virginia; Washington; West Virginia; Wisconsin; Wyoming | 0.05 |
| 2021 | 19 | 305 | 50 | Alabama; Arizona; Arkansas; California; Colorado; Connecticut; Delaware; District of Columbia; Florida; Georgia; Hawaii; Idaho; Illinois; Indiana; Iowa; Kansas; Kentucky; Louisiana; Maine; Maryland; Massachusetts; Michigan; Minnesota; Mississippi; Missouri; Nebraska; Nevada; New Hampshire; New Jersey; New Mexico; New York; North Carolina; North Dakota; Ohio; Oklahoma; Oregon; Pennsylvania; Puerto Rico; Rhode Island; South Carolina; South Dakota; Tennessee; Texas; Utah; Vermont; Virginia; Washington; West Virginia; Wisconsin; Wyoming | 0.06 |
| 2022 | 16 | 341 | 52 | Alabama; Alaska; Arizona; Arkansas; California; Colorado; Connecticut; Delaware; District of Columbia; Florida; Georgia; Hawaii; Idaho; Illinois; Indiana; Iowa; Kansas; Kentucky; Louisiana; Maine; Maryland; Massachusetts; Michigan; Minnesota; Mississippi; Missouri; Montana; Nebraska; Nevada; New Hampshire; New Jersey; New Mexico; New York; North Carolina; North Dakota; Ohio; Oklahoma; Oregon; Pennsylvania; Puerto Rico; Rhode Island; South Carolina; South Dakota; Tennessee; Texas; Utah; Vermont; Virginia; Washington; West Virginia; Wisconsin; Wyoming | 0.06 |
