# Supplementary material for: Multistate animal-contact-related nontyphoidal Salmonella enterica outbreaks in the United States, 2009–2022: Network and machine learning analyses of exposure sources, settings, and serovars
Source: PLoS One. 2026 Jun 5;21(6):e0344889. doi: 10.1371/journal.pone.0344889 (PMC13240899; doi:10.1371/journal.pone.0344889)
Supplement: S2 Table — The output from which the appropriate model was selected for the graph by the software based on BIC and AIC values. APC (Annual Percent Change), Segment Start (Point where Joinpoint is inserted), Segment end (where next Joinpoint is inserted or trend stabilized), APC 95% LCL (Annual Percent Change 95% Lower Confidence Limit), APC 95% UCL (APC 95% Upper Confidence Limit). The Joinpoint regression analysis presents different models with joinpoints, significant and nonsignificant temporal shifts over time. (DOCX) [file pone.0344889.s002.docx]

**S2 Table. Trend analysis results of the Joinpoint regression models of nontyphoidal *Salmonella enterica* multistate outbreak incidence rates at the national level across the U.S., 2009 – 2022**

| **Country** | **Model** | **Joinpoint** | **Segment Start** | **Segment End** | **APC** | **APC 95% LCL** | **APC 95% UCL** | **APC Significant** | **P-Value** |
| --- | --- | --- | --- | --- | --- | --- | --- | --- | --- |
| United States | 0 | 0 | 2009 | 2022 | -2.75 | -4.59 | -0.86 | 1 | 0.008 |
|  | 1 | 0 | 2009 | 2013 | -10.44 | -23.56 | -4.20 | 1 | 0.000 |
|  | 1 | 1 | 2013 | 2022 | -0.27 | -2.39 | 6.76 | 0 | 0.982 |
|  | 2 | 0 | 2009 | 2013 | -12.04 | -17.72 | -5.96 | 1 | 0.003 |
|  | 2 | 1 | 2013 | 2018 | 2.44 | -4.04 | 9.35 | 0 | 0.402 |
|  | 2 | 2 | 2018 | 2022 | -4.46 | -10.63 | 2.14 | 0 | 0.146 |

The output from which the appropriate model was selected for the graph by the software based on BIC and AIC values.APC (Annual Percent Change), Segment Start (Point where Joinpoint is inserted), Segment end (where next Joinpoint is inserted or trend stabilized), APC 95% LCL (Annual Percent Change 95% Lower Confidence Limit), APC 95% UCL (APC 95% Upper Confidence Limit). The Joinpoint regression analysis presents different models with joinpoints, significant and nonsignificant temporal shifts over time.
