# Supplementary material for: Multistate animal-contact-related nontyphoidal Salmonella enterica outbreaks in the United States, 2009–2022: Network and machine learning analyses of exposure sources, settings, and serovars
Source: PLoS One. 2026 Jun 5;21(6):e0344889. doi: 10.1371/journal.pone.0344889 (PMC13240899; doi:10.1371/journal.pone.0344889)
Supplement: S3 Table — The above table indicates the Random Forest (RF) classifier ranking of categorical variables. Each category’s rank (relative importance) has been mentioned. (DOCX) [file pone.0344889.s003.docx]

**S3 Table. Random Forest classifier ranking (relative importance) of categorical variables in nontyphoidal *Salmonella enterica* animal contact-related multistate outbreaks across the US., 2009 – 2022**

| **Variable** | **Abbreviation** | **Category** | **Type** | **Rank** |
| --- | --- | --- | --- | --- |
| Ohio | OH | State | Categorical | 1st |
| Nebraska | NE | State | Categorical | 2nd |
| Indiana | IN | State | Categorical | 3rd |
| Vermont | VT | State | Categorical | 4th |
| Minnesota | MN | State | Categorical | 5th |
| Pennsylvania | PA | State | Categorical | 6th |
| Idaho | ID | State | Categorical | 7th |
| Oregon | OR | State | Categorical | 8th |
| Michigan | MI | State | Categorical | 9th |
| Texas | TX | State | Categorical | 10th |
| Kentucky | KY | State | Categorical | 11th |
| Alabama | AL | State | Categorical | 12th |
| Illinois | IL | State | Categorical | 13th |
| Colorado | CO | State | Categorical | 14th |
| Utah | UT | State | Categorical | 15th |
| Missouri | MO | State | Categorical | 16th |
| Oklahoma | OK | State | Categorical | 17th |
| Massachusetts | MA | State | Categorical | 18th |
| Maryland | MD | State | Categorical | 19th |
| Virginia | VA | State | Categorical | 20th |
| Georgia | GA | State | Categorical | 21st |
| Iowa | IA | State | Categorical | 22nd |
| Connecticut | CT | State | Categorical | 23rd |
| Louisiana | LA | State | Categorical | 24th |
| Wisconsin | WI | State | Categorical | 25th |
| Washington | WA | State | Categorical | 26th |
| Tennessee | TN | State | Categorical | 27th |
| Kansas | KS | State | Categorical | 28th |
| Maine | ME | State | Categorical | 29th |
| California | CA | State | Categorical | 30th |
| Arizona | AZ | State | Categorical | 31st |
| Wyoming | WY | State | Categorical | 32nd |
| Florida | FL | State | Categorical | 33rd |
| Arkansas | AR | State | Categorical | 34th |
| Mississippi | MS | State | Categorical | 35th |
| Montana | MT | State | Categorical | 36th |
| Nevada | NV | State | Categorical | 37th |
| Delaware | DE | State | Categorical | 38th |
| Hawaii | HI | State | Categorical | 39th |
| Alaska | AK | State | Categorical | 40th |
| Birds | BRD | Animal Sources | Categorical | 1st |
| Reptiles | RPT | Animal Sources | Categorical | 2nd |
| Mammals | MAM | Animal Sources | Categorical | 3rd |
| Agricultural Feed Stores | AFS | Exposure Settings | Categorical | 1st |
| Residential Settings | RSH | Exposure Settings | Categorical | 2nd |
| Dairy/agricultural farm settings | FAR | Exposure Settings | Categorical | 3rd |

The above table indicates the Random Forest (RF) classifier ranking of categorical variables. Each category’s rank (relative importance) has been mentioned.
