# Supplementary material for: Multistate animal-contact-related nontyphoidal Salmonella enterica outbreaks in the United States, 2009–2022: Network and machine learning analyses of exposure sources, settings, and serovars
Source: PLoS One. 2026 Jun 5;21(6):e0344889. doi: 10.1371/journal.pone.0344889 (PMC13240899; doi:10.1371/journal.pone.0344889)
Supplement: S4 Table — The co-occurrence network analysis of NTS multistate outbreaks presents the US states, NTS serovars, animal exposure sources, and settings as nodes. Degree is the number of connections each node has, strength is the frequency of shared outbreaks, Betweenness: the number of times a node is on the shortest path between two nodes, Closeness: how close the node is to all other nodes in the network, Clustering Coefficient: a measurement of local connectivity, and Louvain Communities: a group of densely connected nodes. States: NH-New Hampshire, NJ-New Jersey, NM-New Mexico, NY-New York, NC-North Carolina, ND-North Dakota, RI-Rhode Island, SC-South Carolina, SD-South Dakota, WV-West Virginia, DC-District of Columbia. Animal Sources: FSH-Fish, Exposure settings; OTH-Others, HOS-Hospital, VET-Veterinary clinic, FRG-Fairground, Serovars; Serovars: SPBT-Paratyphi B var. L(+) tartrate + , STP-Saintpaul, SJAV-Javiana,STLK-Telelkebir, UNK-unknown, SHDL-Heidelberg, SHFD-Hartford, SDRB-Durban, SGMN-Gaminara, SLML-Lomalinda, SSTN-Stanley, SHVN-Havana, SLIT-Litchfield, SAPP-Apapa, SFLU-Fluntern, SAGO-Agona, SOFF-Offa, SOTH-other, SALA-Alachua, SANA-Anatum, SMAN-Manhattan, SORA-Oranienburg, SGUN-Guinea, SLIV-Liverpool, SEAL-Ealing, SBAN-Banana, SVIT-Vitkin, SLOM-Lome, SSB3-Subspecies IIIb. (DOCX) [file pone.0344889.s004.docx]

**S4 Table. Nontyphoidal *Salmonella enterica* serovars animal contact-related multistate outbreaks co-occurrence network and Louvain community memberships of nodes with no node metrics across the U.S. 2009 – 2022**

| **Node** | **Label** | **Type** | **Degree** | **Strength** | **Betweenness** | **Closeness** | **Clustering Coefficient** | **Louvain Community** |
| --- | --- | --- | --- | --- | --- | --- | --- | --- |
| NH | New Hampshire | State | 0 | 0 | 0 | 0 | 0 | 3 |
| NJ | New Jersey | State | 0 | 0 | 0 | 0 | 0 | 4 |
| NM | New Mexico | State | 0 | 0 | 0 | 0 | 0 | 5 |
| NY | New York | State | 0 | 0 | 0 | 0 | 0 | 6 |
| NC | North Carolina | State | 0 | 0 | 0 | 0 | 0 | 7 |
| ND | North Dakota | State | 0 | 0 | 0 | 0 | 0 | 8 |
| RI | Rhode Island | State | 0 | 0 | 0 | 0 | 0 | 9 |
| SC | South Carolina | State | 0 | 0 | 0 | 0 | 0 | 10 |
| SD | South Dakota | State | 0 | 0 | 0 | 0 | 0 | 11 |
| WV | West Virginia | State | 0 | 0 | 0 | 0 | 0 | 12 |
| DC | District of Colombia | State | 0 | 0 | 0 | 0 | 0 | 13 |
| FSH | Fish | Source | 0 | 0 | 0 | 0 | 0 | 14 |
| NAA | Not available | Source | 0 | 0 | 0 | 0 | 0 | 15 |
| OTH | Other | Setting | 0 | 0 | 0 | 0 | 0 | 16 |
| HOS | Hospital | Setting | 0 | 0 | 0 | 0 | 0 | 17 |
| VET | Veterinary Clinic | Setting | 0 | 0 | 0 | 0 | 0 | 18 |
| FRG | Fairground | Setting | 0 | 0 | 0 | 0 | 0 | 19 |
| SPBT | Paratyphi B var. L(+) tartrate + | Serovar | 0 | 0 | 0 | 0 | 0 | 20 |
| STP | Saintpaul | Serovar | 0 | 0 | 0 | 0 | 0 | 21 |
| SJAV | Javiana | Serovar | 0 | 0 | 0 | 0 | 0 | 22 |
| STLK | Telelkebir | Serovar | 0 | 0 | 0 | 0 | 0 | 23 |
| UNK | Unknown | Serovar | 0 | 0 | 0 | 0 | 0 | 24 |
| SHDL | Heidelberg | Serovar | 0 | 0 | 0 | 0 | 0 | 25 |
| SHFD | Hartford | Serovar | 0 | 0 | 0 | 0 | 0 | 26 |
| SDRB | Durban | Serovar | 0 | 0 | 0 | 0 | 0 | 27 |
| SGMN | Gaminara | Serovar | 0 | 0 | 0 | 0 | 0 | 28 |
| SLML | Lomalinda | Serovar | 0 | 0 | 0 | 0 | 0 | 29 |
| SSTN | Stanley | Serovar | 0 | 0 | 0 | 0 | 0 | 30 |
| SHVN | Havana | Serovar | 0 | 0 | 0 | 0 | 0 | 31 |

The co-occurrence network analysis of NTS multistate outbreaks presents the US states, NTS serovars, animal exposure sources, and settings as nodes. Degree is the number of connections each node has, strength is the frequency of shared outbreaks, Betweenness: the number of times a node is on the shortest path between two nodes, Closeness: how close the node is to all other nodes in the network, Clustering Coefficient: a measurement of local connectivity, and Louvain Communities: a group of densely connected nodes.

States: NH-New Hampshire, NJ-New Jersey, NM-New Mexico, NY-New York, NC-North Carolina, ND-North Dakota, RI-Rhode Island, SC-South Carolina, SD-South Dakota, WV-West Virginia, DC-District of Columbia. Animal Sources: FSH-Fish, Exposure settings; OTH-Others, HOS-Hospital, VET-Veterinary clinic, FRG-Fairground, Serovars; Serovars: SPBT-Paratyphi B var. L(+) tartrate +, STP-Saintpaul, SJAV-Javiana,STLK-Telelkebir, UNK-unknown, SHDL-Heidelberg, SHFD-Hartford, SDRB-Durban, SGMN-Gaminara, SLML-Lomalinda, SSTN-Stanley, SHVN-Havana, SLIT-Litchfield, SAPP-Apapa, SFLU-Fluntern, SAGO-Agona, SOFF-Offa, SOTH-other, SALA-Alachua, SANA-Anatum, SMAN-Manhattan, SORA-Oranienburg, SGUN-Guinea, SLIV-Liverpool, SEAL-Ealing, SBAN-Banana, SVIT-Vitkin, SLOM-Lome, SSB3-Subspecies IIIb
