# Supplementary material for: Multistate animal-contact-related nontyphoidal Salmonella enterica outbreaks in the United States, 2009–2022: Network and machine learning analyses of exposure sources, settings, and serovars
Source: PLoS One. 2026 Jun 5;21(6):e0344889. doi: 10.1371/journal.pone.0344889 (PMC13240899; doi:10.1371/journal.pone.0344889)
Supplement: Attachment — Submitted filename: comments and suggestions-PONE-D-26-09918-v3.pdf [file pone.0344889.s005.pdf]

Manuscript title [PONE-D-26-09918]: Multistate Animal-Contact-Related Nontyphoidal Salmonella enterica Outbreaks in the United States, 2009-2022: Network and Machine Learning Analyses of Exposure Sources, Settings, and Serovars

Overall comment: The authors conducted a study titled "*Multistate Animal-Contact-Related Nontyphoidal Salmonella enterica Outbreaks in the United States, 2009-2022: Network and Machine Learning Analyses of Exposure Sources, Settings, and Serovars*" that analyzed animal contact-related non-typhoidal Salmonella (NTS) outbreak data, which was utilized for calculating incidence rate per 10 million population-years, assessed temporal trends using Join point regression, co-occurrence network linking, and machine learning classification algorithm, such as a random forest classifier to identify variables most useful for distinguishing outbreak profiles. Overall, the manuscript is well written and provides important information on the burden of multistate NTS outbreaks associated with animal contact. This manuscript addresses an important one-health issue. However, the following comments need to be addressed to improve the manuscript clarity and readability.

Specific comment:

Introduction:

Comment: The author should include a brief introduction about the public health burden of non-typhoidal Salmonella (NTS), including the predominant NTS serovars in the United States.

Comment: The relevance of the study is unclear. The author should clarify why this study findings are important (by addressing the "so what?" question in the introduction section).

Materials and Methods:

Comment: The author should provide a brief description of how animal contact related NTS outbreak surveillance data were collected to help the reader understand the data source, collection, population, and reporting process.

Comment: The author should describe how the surveillance data is assessed, including missing values, value distributions, and duplication, before preparing the analytic dataset for this study.

Comment: Authors should provide a clear case definition of 'animal contact related NTS outbreak' for better understanding by the readers of the paper. Additionally, the author should mention the inclusion criteria for including 'animal contact related NTS outbreak' events for analysis in this study.

Comment: The author should report the study design in the methods section.

Comment (Line 116): The author should provide a full elaboration of 'NORS' before introducing the abbreviation.

Comment (Line 145): Authors should provide a table that displays all explanatory variables (features), along with their categories and types, to enhance reader understanding about the variables of interest in this study.

Comment: The author should provide a logical order of data analysis, with their headings, to test each hypothesis in this study

Comment: The author should specify the version of the Joinpoint Regression (JPR) software used in this study.

Comment (Line 144): What is the reasoning behind exclusively using the random forest model (RF)? How do the authors partition (%) the data into training and testing sets for the RF model? The author should provide this information. Did the author try any alternative classification algorithm (e.g., logistic regression ….) as part of the sensitivity analysis? What form of cross-validation was used to assess the RF model's performance? The author should specify the R packages used for the RF modeling.

Comment: The author should provide a brief introduction and a rationale for the network analysis and explain why the network is treated as undirected and weighted. Additionally, the author should specify the R packages used in the study's network analysis.

Comment (Line 144): In the RF model section, the author should specify the outcome variable and explain how it is defined. Also, specify the predictor of interest and explain how the predictors were chosen for inclusion in the model.

Comment: It would be helpful if the author included the full R scripts for the RF model and network analyses as supplementary files to help readers and learners understand the analyses.

Results:

Comment (Line 190-192): These sentences should be in the method section, not part of the result section of the manuscript.

Comment (Line 193-194): These sentences should be placed in the discussion section, not in the results section. In the RF model results, the reader can expect to see the RF model performance matrices and the variable importance identified in the RF model (top, second, and third variables) as the strongest predictors of the outcome variable of interest.

Comment (Line 228-232): These sentences are not results; they are part of the discussion. The author should remove this section from the results section and place it in the appropriate section in the discussion.

Discussion

Comment: The author should provide a primary conclusion statement that logically and defensibly draws from the study results.
