# Supplementary material for: Multistate animal-contact-related nontyphoidal Salmonella enterica outbreaks in the United States, 2009–2022: Network and machine learning analyses of exposure sources, settings, and serovars
Source: PLoS One. 2026 Jun 5;21(6):e0344889. doi: 10.1371/journal.pone.0344889 (PMC13240899; doi:10.1371/journal.pone.0344889)
Supplement: Attachment — Submitted filename: Response to Editor_Reviewers.R1.docx [file pone.0344889.s006.docx]

**PLOS One**

**PONE-D-26-09918**

**Multistate Animal-Contact-Related Nontyphoidal *Salmonella enterica* Outbreaks in the United States, 2009-2022: Network and Machine Learning Analyses of Exposure Sources, Settings, and Serovars**

We are thankful to the editor and reviewers for their constructive feedback. We have addressed all comments to improve the organization, clarity, and transparency of our manuscript. The revisions have been incorporated into the updated manuscript, as described below.

**Response to Journal Requirements**

Journal requirements:

When submitting your revision, we need you to address these additional requirements.

**Comment**

**1. Please ensure that your manuscript meets PLOS ONE's style requirements, including those for file naming. The PLOS ONE style templates can be found at**

[**https://journals.plos.org/plosone/s/file?id=wjVg/PLOSOne_formatting_sample_main_body.pdf**](https://urldefense.com/v3/__https:/journals.plos.org/plosone/s/file?id=wjVg*PLOSOne_formatting_sample_main_body.pdf__;Lw!!DZ3fjg!9TRm7nWY1OOcYx3iSiBkHjf15ewIxwWN18CNRmdhFvCoFm7HXEQPFAPF2coF7KVD9cghsxtzK0-j4ikfdA$) **and**

[**https://journals.plos.org/plosone/s/file?id=ba62/PLOSOne_formatting_sample_title_authors_affiliations.pdf**](https://urldefense.com/v3/__https:/journals.plos.org/plosone/s/file?id=ba62*PLOSOne_formatting_sample_title_authors_affiliations.pdf__;Lw!!DZ3fjg!9TRm7nWY1OOcYx3iSiBkHjf15ewIxwWN18CNRmdhFvCoFm7HXEQPFAPF2coF7KVD9cghsxtzK0_WG3TfRg$)

**Response:**

The manuscript follows PLOS ONE's style requirements as outlined in the provided templates. Please see the revised manuscript file.

2. **Please note that PLOS One has specific guidelines on code sharing for submissions in which author-generated code underpins the findings in the manuscript. In these cases, we expect all author-generated code to be made available without restrictions upon publication of the work. Please review our guidelines at** [**https://journals.plos.org/plosone/s/materials-and-software-sharing#loc-sharing-code**](https://urldefense.com/v3/__https:/journals.plos.org/plosone/s/materials-and-software-sharing*loc-sharing-code__;Iw!!DZ3fjg!9TRm7nWY1OOcYx3iSiBkHjf15ewIxwWN18CNRmdhFvCoFm7HXEQPFAPF2coF7KVD9cghsxtzK0_AqJQcZA$) **and ensure that your code is shared in a way that follows best practice and facilitates reproducibility and reuse.**

**Response:**

The codes used in the analysis will be shared upon acceptance of our work.

**Comment**

**3. Please include captions for your Supporting Information files at the end of your manuscript, and update any in-text citations to match accordingly. Please see our Supporting Information guidelines for more information:** [**http://journals.plos.org/plosone/s/supporting-information**](https://urldefense.com/v3/__http:/journals.plos.org/plosone/s/supporting-information__;!!DZ3fjg!9TRm7nWY1OOcYx3iSiBkHjf15ewIxwWN18CNRmdhFvCoFm7HXEQPFAPF2coF7KVD9cghsxtzK09WVZdVZA$).

**Response:**

The in-text citations were updated according to PLOS ONE requirements. The supporting information file has also been updated accordingly.

**4. We note that Figure 4 in your submission contain [map/satellite] images which may be copyrighted. All PLOS content is published under the Creative Commons Attribution License (CC BY 4.0), which means that the manuscript, images, and Supporting Information files will be freely available online, and any third party is permitted to access, download, copy, distribute, and use these materials in any way, even commercially, with proper attribution. For these reasons, we cannot publish previously copyrighted maps or satellite images created using proprietary data, such as Google software (Google Maps, Street View, and Earth). For more information, see our copyright guidelines:** [**http://journals.plos.org/plosone/s/licenses-and-copyright**](https://urldefense.com/v3/__http:/journals.plos.org/plosone/s/licenses-and-copyright__;!!DZ3fjg!9TRm7nWY1OOcYx3iSiBkHjf15ewIxwWN18CNRmdhFvCoFm7HXEQPFAPF2coF7KVD9cghsxtzK09yfPM01w$)**.**

**We require you to either (1) present written permission from the copyright holder to publish these figures specifically under the CC BY 4.0 license, or (2) remove the figures from your submission:**

**1. You may seek permission from the original copyright holder of Figure 4 to publish the content specifically under the CC BY 4.0 license.**

**We recommend that you contact the original copyright holder with the Content Permission Form (**[**http://journals.plos.org/plosone/s/file?id=7c09/content-permission-form.pdf**](https://urldefense.com/v3/__http:/journals.plos.org/plosone/s/file?id=7c09*content-permission-form.pdf__;Lw!!DZ3fjg!9TRm7nWY1OOcYx3iSiBkHjf15ewIxwWN18CNRmdhFvCoFm7HXEQPFAPF2coF7KVD9cghsxtzK08kQzbUEw$)**) and the following text:**

**“I request permission for the open-access journal PLOS ONE to publish XXX under the Creative Commons Attribution License (CCAL) CC BY 4.0 (**[**http://creativecommons.org/licenses/by/4.0/**](https://urldefense.com/v3/__http:/creativecommons.org/licenses/by/4.0/__;!!DZ3fjg!9TRm7nWY1OOcYx3iSiBkHjf15ewIxwWN18CNRmdhFvCoFm7HXEQPFAPF2coF7KVD9cghsxtzK08GSb69XQ$)**). Please be aware that this license allows unrestricted use and distribution, even commercially, by third parties. Please reply and provide explicit written permission to publish XXX under a CC BY license and complete the attached form.”**

**Please upload the completed Content Permission Form or other proof of granted permissions as an "Other" file with your submission.**

**In the figure caption of the copyrighted figure, please include the following text: “Reprinted from [ref] under a CC BY license, with permission from [name of publisher], original copyright [original copyright year].”**

**Response:**

The base maps were generated using TIGER/Line shapefiles provided by the United States Census Bureau. These are in the public domain and not subject to copyright. Under the US copyright act (USC § 105), works of the US government are not eligible for copyright protection. Therefore, the maps are freely available for public use and comply with the CC BY 4.0 licensing requirements. Additionally, we have indicated this in our data source section of materials and methods.

**2. If you are unable to obtain permission from the original copyright holder to publish these figures under the CC BY 4.0 license or if the copyright holder’s requirements are incompatible with the CC BY 4.0 license, please either i) remove the figure or ii) supply a replacement figure that complies with the CC BY 4.0 license. Please check copyright information on all replacement figures and update the figure caption with source information. If applicable, please specify in the figure caption text when a figure is similar but not identical to the original image and is therefore for illustrative purposes only.**

**The following resources for replacing copyrighted map figures may be helpful:**

**USGS National Map Viewer (public domain):** [**http://viewer.nationalmap.gov/viewer/**](https://urldefense.com/v3/__http:/viewer.nationalmap.gov/viewer/__;!!DZ3fjg!9TRm7nWY1OOcYx3iSiBkHjf15ewIxwWN18CNRmdhFvCoFm7HXEQPFAPF2coF7KVD9cghsxtzK0-TA15ccQ$)

**The Gateway to Astronaut Photography of Earth (public domain):** [**http://eol.jsc.nasa.gov/sseop/clickmap/**](https://urldefense.com/v3/__http:/eol.jsc.nasa.gov/sseop/clickmap/__;!!DZ3fjg!9TRm7nWY1OOcYx3iSiBkHjf15ewIxwWN18CNRmdhFvCoFm7HXEQPFAPF2coF7KVD9cghsxtzK0-UpfvfrQ$)

**Maps at the CIA (public domain):** [**https://www.cia.gov/library/publications/the-world-factbook/index.html**](https://urldefense.com/v3/__https:/www.cia.gov/library/publications/the-world-factbook/index.html__;!!DZ3fjg!9TRm7nWY1OOcYx3iSiBkHjf15ewIxwWN18CNRmdhFvCoFm7HXEQPFAPF2coF7KVD9cghsxtzK08COXe1wA$) **and** [**https://www.cia.gov/library/publications/cia-maps-publications/index.html**](https://urldefense.com/v3/__https:/www.cia.gov/library/publications/cia-maps-publications/index.html__;!!DZ3fjg!9TRm7nWY1OOcYx3iSiBkHjf15ewIxwWN18CNRmdhFvCoFm7HXEQPFAPF2coF7KVD9cghsxtzK0_9uNc0rA$)

**NASA Earth Observatory (public domain):** [**http://earthobservatory.nasa.gov/**](https://urldefense.com/v3/__http:/earthobservatory.nasa.gov/__;!!DZ3fjg!9TRm7nWY1OOcYx3iSiBkHjf15ewIxwWN18CNRmdhFvCoFm7HXEQPFAPF2coF7KVD9cghsxtzK0_9CnZKKw$)

**Landsat:** [**http://landsat.visibleearth.nasa.gov/**](https://urldefense.com/v3/__http:/landsat.visibleearth.nasa.gov/__;!!DZ3fjg!9TRm7nWY1OOcYx3iSiBkHjf15ewIxwWN18CNRmdhFvCoFm7HXEQPFAPF2coF7KVD9cghsxtzK0-fVm0dyQ$)

**USGS EROS (Earth Resources Observatory and Science (EROS) Center) (public domain):** [**http://eros.usgs.gov/#**](https://urldefense.com/v3/__http:/eros.usgs.gov/*__;Iw!!DZ3fjg!9TRm7nWY1OOcYx3iSiBkHjf15ewIxwWN18CNRmdhFvCoFm7HXEQPFAPF2coF7KVD9cghsxtzK0-8qcHOkw$)

**Natural Earth (public domain):** [**http://www.naturalearthdata.com/**](https://urldefense.com/v3/__http:/www.naturalearthdata.com/__;!!DZ3fjg!9TRm7nWY1OOcYx3iSiBkHjf15ewIxwWN18CNRmdhFvCoFm7HXEQPFAPF2coF7KVD9cghsxtzK09_K-dd_w$)

**Response:**

Not applicable to our situation. Please see our response above.

**5. In the online submission form, you indicated that [Data cannot be shared publicly because of data protection. Data are available from the CDC NORS via data request.].**

**All PLOS journals now require all data underlying the findings described in their manuscript to be freely available to other researchers, either 1. In a public repository, 2. Within the manuscript itself, or 3. Uploaded as supplementary information.**

**This policy applies to all data except where public deposition would breach compliance with the protocol approved by your research ethics board. If your data cannot be made publicly available for ethical or legal reasons (e.g., public availability would compromise patient privacy), please explain your reasons on resubmission and your exemption request will be escalated for approval.**

**If the reviewer comments include a recommendation to cite specific previously published works, please review and evaluate these publications to determine whether they are relevant and should be cited. There is no requirement to cite these works unless the editor has indicated otherwise.**

[Note: HTML markup is below. Please do not edit.]

**Response:**

We updated the data availability statement as follows: “The data underlying the findings described in the manuscript are available within the manuscript and in the supplementary files.“

**Response to Reviewers**

**The author should include a brief introduction about the public health burden of non-typhoidal Salmonella (NTS), including the predominant NTS serovars in the United States.**

**Response:**

Thank you for your suggestion. We included a section on the disease burden and main NTS serovars:

“Globally, NTS causes over 95 million enteric illnesses, with more than 500,000 invasive infections, leading to high fatality rates [2]. It also poses an economic burden, contributing to millions of disability-adjusted life years and to substantial healthcare costs worldwide [3]. Most of NTS infections are self-limiting; however, severe invasive disease can occur, primarily affecting immunocompromised individuals, infants, children, and the elderly, often resulting in death [4]. The NTS bacteria are adaptable to various hosts, with NTS serovars such as Typhimurium, Enteritidis, Heidelberg, and Infantis being common causes of human enteric disease, including in the U.S. [5]. “

**Comment:**

**The relevance of the study is unclear. The author should clarify why this study findings are important (by addressing the “so what?” question in the introduction section).**

**Response:**

We updated the introduction section to address your comment and included the following:

“No previous study explored long-term, NTS serovar-level animal contact-related multistate outbreaks in the US, and the relationship between serovars and animal contact sources remains unclear. Previous epidemiological studies focused on individual serovars or specific exposure sources, limiting the full scope of NTS transmission dynamics across the U.S. [16,21,22]. In multistate outbreaks, interconnected events might involve shared distribution systems, supply chains, and occupational exposures [21], and an integrated analytical approach is needed to quantify NTS burden and identify factors that sustain its endemicity across various reservoirs and regions.”

**Comment:**

**The author should provide a brief description of how animal contact related NTS outbreak surveillance data were collected to help the reader understand the data source, collection, population, and reporting process.**

**Response:**

Thank you for your suggestions.

WE included a section on NORS and included the following:

“**Data source, study design, and setting**

The National Outbreak Reporting System (NORS) is a voluntary disease surveillance system managed by the Centers for Disease Control and Prevention (CDC) that collects reports on enteric disease outbreaks, including those transmitted through animal contact. Animal contact is defined as any interaction between humans and animals that may result in the transmission of infectious agents. This includes direct contact with animals, such as handling, feeding, or caring for them, and indirect contact through exposure to animal environments, such as barns, petting zoos, or farms. Exposure sources include livestock, poultry, reptiles, and other animals, and outbreaks are included in this category where animal contact is suspected or confirmed as a contributing factor to the transmission of infectious diseases. [23, 24]. The NORS is used by local health departments, state-level health authorities, and the CDC to report and track outbreaks [24]. An outbreak is defined as the occurrence of more cases than expected within a period, a specific location, and a target population [25]. Multistate (more than 2 states) NTS outbreak investigations are coordinated and reported to NORS by CDC staff, while single-state outbreaks are investigated and reported to NORS by single jurisdictions [26].”

**Comment:**

**The author should describe how the surveillance data is assessed, including missing values, value distributions, and duplication, before preparing the analytic dataset for this study.**

**Response:**

We included all the available data in NORS. To explain our study approach, we included the following: This retrospective observational study analyzes human NTS animal contact-related multistate outbreaks. It includes all outbreaks with confirmed or suspected status, along with data on serovars, exposure states, animal contact exposure sources, and settings. Outbreaks with “not available” or "other" categories for serovars, exposure sources, and settings were treated as distinct categories for further analysis.”

**Comment:**

**Authors should provide a clear case definition of 'animal contact related NTS outbreak' for better understanding by the readers of the paper. Additionally, the author should mention the inclusion criteria for including ‘animal contact related NTS outbreak' events for analysis in this study.**

**Response:**

Thank you for your comment. We included the definition of “animal-contact outbreaks, and included the following:

“Animal contact is defined as any interaction between humans and animals that may result in the transmission of infectious agents. This includes direct contact with animals, such as handling, feeding, or caring for them, and indirect contact through exposure to animal environments, such as barns, petting zoos, or farms. Exposure sources include livestock, poultry, reptiles, and other animals, and outbreaks are included in this category where animal contact is suspected or confirmed as a contributing factor to the transmission of infectious diseases.”

**Comment:**

**The author should report the study design in the methods section.**

**Response:**

We included the following: “This retrospective observational study analyzes human NTS animal contact-related multistate outbreaks.”

**Comment:**

**(Line 116): The author should provide a full elaboration of ‘NORS’ before introducing the abbreviation.**

**Response:**

We corrected this and defined NORS before the abbreviation.

**Comment:**

**(Line 145): Authors should provide a table that displays all explanatory variables (features), along with their categories and types, to enhance reader understanding about the variables of interest in this study.**

**Response:**

Thank you for your suggestion. We included a Table at the end of the Descriptive Statistics section to describe the variables used in our analysis.

We included the following: “Table 1 summarizes the explanatory variables (features) used in the analysis, including their description, type, category, and the specific analysis in which they were applied.

**Table 1. Summary of variables used for the analysis of Nontyphoidal Salmonella enterica animal contact-related multistate outbreaks across the US, 2009 – 2022**“

**Comment:**

**The author should provide a logical order of data analysis, with their headings, to test each hypothesis in this study**

**Response:**

We provided a logical data analysis starting from general and more complex analyses. We included subsection titles for each step, as follows:

Assessing NTS outbreak incidence rates

Temporal trend analysis using the Joinpoint regression method

Random Forest analysis of multi-state outbreak data

Network analysis of multi-state outbreak data

**Comment:**

**The author should specify the version of the Joinpoint Regression (JPR) software used in this study.**

**Response:**

The version of the Joinpoint Regression software was added.

**Comment**

**(Line 144): What is the reasoning behind exclusively using the random forest model (RF)?**

**Response**

We have used the RF model as it can handle sparse reported binary surveillance data and to learn the nonlinear relationships amongst the different feature variables embedded into the model. We updated the manuscript, and included the following: “We analyzed the binary dataset of human NTS multistate outbreaks from 2009 to 2022 using the Random Forest (RF) modeling approach. The RF method is a flexible, non-parametric ensemble learning technique that can perform well with high-dimensional datasets, which contain correlated and binary predictors, as our surveillance dataset carries the same characteristics. The RF method can also model complex, non-linear relationships among the higher-dimensional model variables, and it can also include higher-order interactions without requiring explicit specifications. In addition, RF also incorporates internally validated performance estimates through out-of-bag (OOB) error and can generate interpretable variable importance measures, which were central to our objective of identifying the key driving factors distinguishing cluster membership into the model [36]. The RF model is less sensitive to multicollinearity and overfitting than traditional regression-based approaches, and is suitable for exploratory analyses of complex multistate outbreak data with many potentially correlated indicators.”

**Comment**

**How do the authors partition (%) the data into training and testing sets for the RF model?**

**Response**

We didn’t implement an explicit training/test split or cross-validation procedure. We have checked the model performance through the out-of-bag (OOB) error estimation inherent to the random forest algorithm; this has now been clarified in the manuscript.

We included the following: “In addition, RF also incorporates internally validated performance estimates throughout out-of-bag (OOB) error and can generate interpretable variable importance measures, which were central to our objective of identifying the key driving factors distinguishing cluster membership into the model “

**Comment**

**The author should provide this information. Did the author try any alternative classification algorithm (e.g., logistic regression ….) as part of the sensitivity analysis?**

**Response**

Logistic regression assumes the linear relationships amongst the variables and is sensitive to multicollinearity, and our dataset potentially includes many correlated binary indicators and potential interactions, and thus making RF more appropriate. Potentially, other classification algorithms can be utilized, but to compare this was beyond the scope of our current manuscript.

**What form of cross‑validation was used to assess the RF model's performance? The author should specify the R packages used for the RF modeling.**

**Response:**

The goal of our work was to find the interpretation variable importance and rank them accordingly, rather than pure prediction, and we have utilized the entire dataset for that. So, we abstain from cross-validation and use the inherent out-of-bag (OOB) error estimation to assess the model performance. We explained this in the section where we describe the RF model. Also, we included the R packages that we used for the RF model.

**Comment:**

**The author should provide a brief introduction and a rationale for the network analysis and explain why the network is treated as undirected and weighted. Additionally, the author should specify the R packages used in the study's network analysis.**

Response:

Thank you for your comments. We included the following: “The co-occurrence network approach was used to characterize relationships (co-occurrences) among NTS serovars, animal-contact exposure sources, settings, and States in multistate outbreaks [45]. This approach enables the identification of co-occurrence patterns and clustering structures across outbreak features. In our study, an undirected network was developed because the outbreak surveillance data showed shared participation in the same outbreak, and no directional transmission pathways could be established. To display the frequency of co-occurrence between categorical variables, a weighted network structure was used to allow for more recurrent relationships within the network [46].”

“We further described this network as weighted, where edge weights represented the magnitude of co-occurrence between node pairs, thus allowing stronger and weaker associations to be distinguished and visually represented.”

“The R packages that were used to explore co-occurrence network patterns were igraph [45], ggraph [48], ggplot2 [43], and networkD3 [47].”

**Comment:**

**In the RF model section, the author should specify the outcome variable and explain how it is defined. Also, specify the predictor of interest and explain how the predictors were chosen for inclusion in the model.**

**Response:**

We have revised the manuscript to clarify the analytical approach. We have used the cluster membership as the outcome variable in the random forest model, and it derived from hierarchical clustering using Jaccard distance and Ward’s method. All surveillance variables representing state, animal source, and exposure setting are included as predictors after excluding invariant variables. We updated the model explanation by including the following:

“We performed unsupervised hierarchical clustering on the binary matrix using Jaccard dissimilarity and Ward’s linkage, and then considered the resulting dendrogram, which was cut into four clusters [38]. We treated the cluster membership as a 4-level categorical outcome in an RF model [39]. The outcome variable in the RF model was the cluster membership of multistate NTS outbreaks, categorized into four distinct groups based on similarities in their predictors (Table 1). In our model exploration, we included all remaining surveillance variables as predictors, and the variable importance was summarized using mean decrease in accuracy and mean decrease in Gini coefficient. Collectively, the above-mentioned steps involved identifying clusters through unsupervised methods, followed by the application of an RF classifier to determine the variables that distinguish cluster membership of the surveillance data (Table 1).“

**Comment:**

**It would be helpful if the author included the full R scripts for the RF model and network analyses as supplementary files to help readers and learners understand the analyses.**

**Response:**

Once the manuscript is accepted, we will include the GitHub link for the R code.

**Comment:**

**(Line 190-192): These sentences should be in the method section, not part of the result section of the manuscript.**

**Response:**

We moved this sentence to the methods section.

**Comment:**

**(Line 193-194): These sentences should be placed in the discussion section, not in the results section. In the RF model results, the reader can expect to see the RF model performance matrices and the variable importance identified in the RF model (top, second, and third variables) as the strongest predictors of the outcome variable of interest.**

**Response:**

We moved this sentence to the discussion section.

**Comment:**

**(Line 228-232): These sentences are not results; they are part of the discussion. The author should remove this section from the results section and place it in the appropriate section in the discussion.**

**Response:**

We moved this sentence to the discussion section.

**Comment:**

**The author should provide a primary conclusion statement that logically and defensibly draws from the study results.**

**Response:**

Thank you for your suggestion. We included a Conclusion section: ”This study examined animal contact-related multistate NTS outbreaks across the U.S. between 2009 and 2022. The study findings suggest that outbreaks in different regions (communities) were associated with distinct serovar profiles, exposure sources, and settings, highlighting potential geographical and environmental factors that drive the spread of NTS infections. Machine learning and network analysis identified key serovars (Enteritidis, Typhimurium, Infantis, Hadar) and exposure sources (birds, reptiles, mammals) and settings ( agricultural feed stores, residential, and farms), in the Midwest, Northeast, and Mountain West, as influential factors shaping outbreak patterns. Temporal trends in the NTS outbreak IRs indicated a stabilizing pattern in later years. These findings highlight the complexity of NTS transmission dynamics across human, animal, and environmental interfaces, which traditional epidemiological methods cannot fully capture. Integrating advanced analytical tools into disease surveillance can enhance understanding of the factors driving NTS persistence and transmission. Future research should refine these models to support public health initiatives aimed at reducing NTS disease burden.“
